# Effect of Myxoma Virus Species Jump on Iberian Hare Populations

**DOI:** 10.3201/eid3006.231280

**Published:** 2024-06

**Authors:** Beatriz Cardoso, Ignacio García-Bocanegra, João Queirós, Javier Fernández-López, Paulo C. Alves, Pelayo Acevedo

**Affiliations:** Centro de Investigação em Biodiversidade e Recursos Genéticos, Vairão, Portugal (B. Cardoso, J. Queirós, P.C. Alves);; Faculdade de Ciências da Universidade do Porto, Oporto, Portugal (B. Cardoso, J. Queirós, P.C. Alves);; Estação Biológica de Mértola, Mértola, Portugal (B. Cardoso, J. Queirós, P.C. Alves);; Instituto de Investigación en Recursos Cinegéticos, Ciudad Real, Spain (B. Cardoso, P. Acevedo);; University of Córdoba, Cordoba, Spain (I. García-Bocanegra);; Centre d’Ecologie Fonctionnelle et Evolutive, Montpellier, France (J. Fernández-López);; Universidad Complutense de Madrid, Madrid, Spain (J. Fernández-López)

**Keywords:** myxoma virus, Iberian hare, Lepus granatensis, population decline, recombinant myxoma virus, Iberian Peninsula, wildlife, disease impact, pathogen emergence, game management, viruses, Portugal, Spain

## Abstract

The myxoma virus species jump from European rabbits (*Oryctolagus cuniculus*) to Iberian hares (*Lepus granatensis*) has raised concerns. We assess the decline suffered by Iberian hare populations on the Iberian Peninsula and discuss the association between the effect of myxomatosis and the average abundance index, which we estimated by using hunting bags.

In July 2018, after 60 years of endemic circulation in European wild rabbits (*Oryctolagus cuniculus*), myxoma virus (MYXV) jumped to the Iberian hare (*Lepus granatensis*) (*1*). This species jump resulted from the emergence of a recombinant strain of MYXV, named ha-MYXV, containing a 2.8-kb insertion derived from an unknown poxvirus (*2*,*3*). Outbreak notifications rapidly spread across the Iberian Peninsula, resulting in an estimated mean mortality rate of 55.4% (median 70%) in hares (*4*). Concerns were raised about the effect of myxomatosis on the Iberian hare populations (*4*). We investigated those concerns and determined how myxomatosis affected Iberian hares by evaluating hare abundance indexes before and after the emergence of ha-MYXV.

We used hunting bag data to approximate population abundance (*5*). We collected information on hunting yields from hunting grounds in Portugal and the most affected regions of Spain, Andalusia, and Castilla-La Mancha during the hunting seasons (October–February) spanning from 2007–08 to 2020–21. Our study period includes 11 seasons before ha-MYXV emergence (premyxomatosis), from 2007–08 to 2017–18, and 3 after (postmyxomatosis), from 2018–19 until 2020–21. For each hunting ground and season, we estimated the abundance index as the number of hares hunted per square kilometer. We used analysis of variance tests to evaluate the differences between abundance indexes. We gathered data on myxomatosis outbreaks from nationwide passive surveillance efforts conducted after the first case reports. We used the coefficient (−1 to 1) obtained from the linear regression between hunting yields and hunting seasons to compute population trends (*6*) for the study period and the premyxomatosis period. Because the postmyxomatosis period was too short to estimate population trends accurately, we calculated the disease effect as the difference between the global and the premyxomatosis trends. We estimated the threshold of premyxomatosis abundance index from which >50% of populations were negatively affected by the disease.

We found a reduction of 77.2% in hares hunted during the study period (Figure, panel A). In the decade preceding the first myxomatosis outbreak, a smooth negative population trend was noted (https://www.intechopen.com/chapters/71640), with a mean annual reduction of 3.2% and a total decline of 29.6% in the number of hunted Iberian hares (Figure, panel A). Coinciding with the emergence of ha-MYXV, the highest annual decline of 51.5% occurred from 2017–18 to 2018–19 (Figure, panel A). This decrease was 57.1% in Andalusia and 50.9% in Castilla-La Mancha. In Portugal, the decrease was only 10.0% but increased to 30.9% in the following hunting season (2019–20). This abrupt population decline could result from the rapid spread of ha-MYXV in the Iberian Peninsula (*4*). The number of hunted hares remained low after 2018, which is not suggestive of a postmyxomatosis recovery (Figure, panel A). Nevertheless, the evolution of hare population trends needs to be monitored over a longer period for more accurate inferences.

**Figure:**
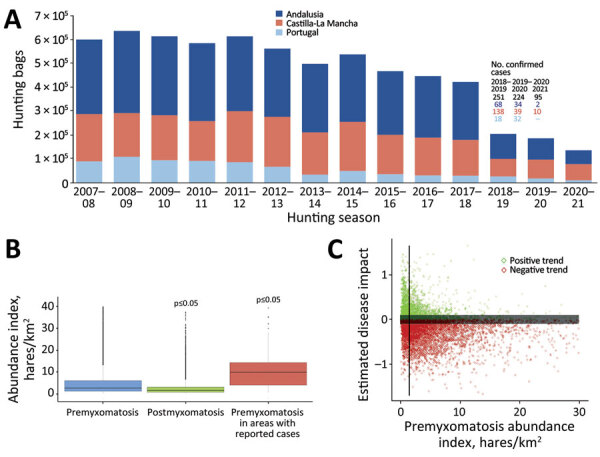
Evidence of the effect of myxomatosis outbreaks on Iberian hare (*Lepus granatensis*) populations and the link to the abundance index, in the Iberian Peninsula, after the initial species jump in 2018. A) Temporal evolution of the hunting yields from 2007–08 to 2020–21, along with the number of confirmed myxomatosis cases per hunting season, in the studied regions. B) Comparison between the average overall abundance index (hunted hares/km^2^) in the premyxomatosis period versus the abundance index estimated for the postmyxomatosis period and the average overall abundance index versus the premyxomatosis period in hunting areas with reported outbreaks. C) Correlation between the estimated effect of myxomatosis (calculated as the difference between global and pre-myxomatosis trends) and the pre-myxomatosis hare abundance index. The vertical line represents the premyxomatosis abundance index threshold (1.5 hunted hares/km^2^) from which most populations were negatively affected by disease. The dark gray buffer zone (trend values between −0.1 and 0.1) comprises hunting grounds excluded to account for the uncertainty of a trend proximate to zero.

We found significant differences (p<0.05) between the mean abundance indexes in the premyxomatosis versus postmyxomatosis periods, demonstrating further evidence of the myxomatosis-related decrease in hare populations (Figure, panel B). Areas with confirmed cases showed higher premyxomatosis abundance indexes compared with the overall average in the same period (Figure, panel B). We found concordant results when investigating the association between the premyxomatosis hare abundance index and the estimated disease effect. We found that, above a threshold of abundance index, the estimated disease effect is likely negative (Figure, panel C). Lower abundances may act as a barrier to virus dispersal, limiting the effect of myxomatosis, as previously described in wild rabbits (*7*). Of note, the abundance index threshold estimated for the study area is low (1.5 hares hunted/km^2^) (Figure, panel C), meaning most hunting grounds have surpassing abundance indexes (76.4% in Spain and 51.0% in Portugal). This finding suggests ha-MYXV is highly effective in establishing itself in Iberian hare populations. The comparatively lower abundance indexes in Portugal may explain the lesser effect of myxomatosis in the Iberian Peninsula region.

The future evolution of myxomatosis in Iberian hare populations is uncertain, and concerns remain if myxomatosis will mimic the evolution documented in European wild rabbits. Hare populations were already in decline during the decade before the first myxomatosis outbreak. Information on hare population status was and still is scarce. To ensure the future sustainability of Iberian hares, long-term and holistic conservation, management, and monitoring programs are needed, especially when worldwide viral emergence events have become increasingly more frequent in lagomorph species over the past decade (*8*,*9*). The conservation status of the Iberian hare across its distribution range should be continuously monitored and reassessed as needed. Our results indicate the decline suffered by Iberian hare populations in the past few years can be linked to the emergence of ha-MYXV.
